# Midgap radiative centers in carbon-enriched hexagonal boron nitride

**DOI:** 10.1073/pnas.2003895117

**Published:** 2020-06-01

**Authors:** Maciej Koperski, Diana Vaclavkova, Kenji Watanabe, Takashi Taniguchi, Kostya S. Novoselov, Marek Potemski

**Affiliations:** ^a^Department of Materials Science and Engineering, National University of Singapore, 117575, Singapore;; ^b^Laboratoire National des Champs Magnétiques Intenses, CNRS–Université Grenoble Alpes–Université Paul Sabatier–Institut National des Sciences Appliquées Toulouse–European Magnetic Field Laboratory, 38042 Grenoble, France;; ^c^Research Center for Functional Materials, National Institute for Materials Science, 1-1 Namiki, Tsukuba 305-0044, Japan;; ^d^International Center for Materials Nanoarchitectonics, National Institute for Materials Science, 1-1 Namiki, Tsukuba 305-0044, Japan;; ^e^Institute of Experimental Physics, Faculty of Physics, University of Warsaw, PL-02-093 Warsaw, Poland

**Keywords:** hexagonal boron nitride, defects, midgap centers, single-photon emitters

## Abstract

When serving as a protection tissue and/or inducing a periodic lateral modulation for/in atomically thin crystals, hexagonal boron nitride (hBN) has revolutionized the research on van der Waals heterostructures. By itself, hBN appears as an emergent wide-bandgap material, which, importantly, can be optically bright in the far-ultraviolet range and which frequently displays midgap defect-related centers of yet-unclear origin, but, interestingly, acting as single-photon emitters. Controlling the hBN doping is of particular interest in view of the possible practical use of this material. Here, we demonstrate that enriching hBN with carbon (C) activates an optical response of this material in the form of a series of well-defined resonances in visible and near-infrared regions, which appear in the luminescence spectra measured under below-bandgap excitation. Two, qualitatively different, C-related radiative centers are identified: One follows the Franck–Condon principle that describes transitions between two defect states with emission/annihilation of optical phonons, and the other shows atomic-like resonances characteristic of intradefect transitions. With a detailed characterization of the energy structure and emission dynamics of these radiative centers, we contribute to the development of controlled doping of hBN with midgap centers.

## Significance

Well‐defined defect centers play a crucial role in condensed-matter physics. That is particularly evident in the case of semiconductors and wide‐gap insulators, where midgap levels provide a number of versatile functionalities: from lasing to pressure sensing. Individual defects may become single-photon sources—crucial for applications in quantum technology. Recently, such defects have been observed in hexagonal boron nitride (hBN). Here, a method to fabricate stable and reproducible defects in hBN is introduced. Large bandgap of boron nitride allows the use of such defects in many quantum applications. Fabrication and characterization of such defects in hBN, which has become a relevant, emerging, wide-bandgap 2D material, is an important step toward novel functionalities of van der Waals heterostructures.

Well-defined defect centers play a crucial role in condensed-matter physics and material science. That is particularly evident in case of semiconductors and wide-gap insulators, where midgap levels may arise due to various imperfections of the crystal lattice. These additional states may manifest themselves in many ways, e.g., giving rise to photoluminescence (1) or participating in charge-tunneling processes (2). They may also provide versatile functionalities, ranging from lasing (3) to pressure sensing (4, 5). When properly isolated and controlled, individual defects may become single-photon sources (6) or building blocks in devices relevant for metrology in nanoscale (7).

One of the materials with interesting, yet incompletely understood, defect centers is hexagonal boron nitride (hBN). Due to a large value of the bandgap (8), equal to 5 eV, the optical response of hBN crystals may span a very broad spectral range (9). Indeed, a deep-ultraviolet (deep-UV) interband photoluminescence (10, 11) is known to be accompanied by 4.2-eV defect-related emission resonances (12), as well as visible (reaching near-infrared wavelengths) spectrally narrow emission lines (13–18). The two latter emitters have been demonstrated to be capable of single-photon emission, but their microscopic origin remains unclear. For the sake of a comprehensive understanding, the atomistic structure of these defects needs to be identified. Also, from practical perspectives, the methods of their controlled creation should be developed. One of the most plausible candidates to account for these defect centers, according to predictions of low-formation energy and large migration barriers (19), are carbon (C)-related impurities. In this work, we explore this hypothesis by inspecting C-doped hBN (hBN:C) crystals, uncovering their robust and intricate optical response in the visible and near-infrared spectral range.

For the purpose of a comparative study of pristine and C-doped samples, hBN single crystals were grown by a high-pressure, temperature-gradient method. These specimens are known to exhibit very low defect density, and their characterization by photoluminescence and/or cathodoluminescence usually does not show any substantial signatures of defect-related light emission. Several hBN crystals originating from the same growth experiment were placed in a graphite furnace and annealed at high temperature. This procedure had an immediately observable impact on the hBN crystals. They changed their appearance from colorless and transparent to yellow, hence providing a first indication that C is incorporated in the hBN material. In order to characterize these crystals through optical methods, we isolated layers of various thicknesses via standard techniques of mechanical exfoliation and deposited them onto ultraflat silicon substrates.

**Fig. 1.**
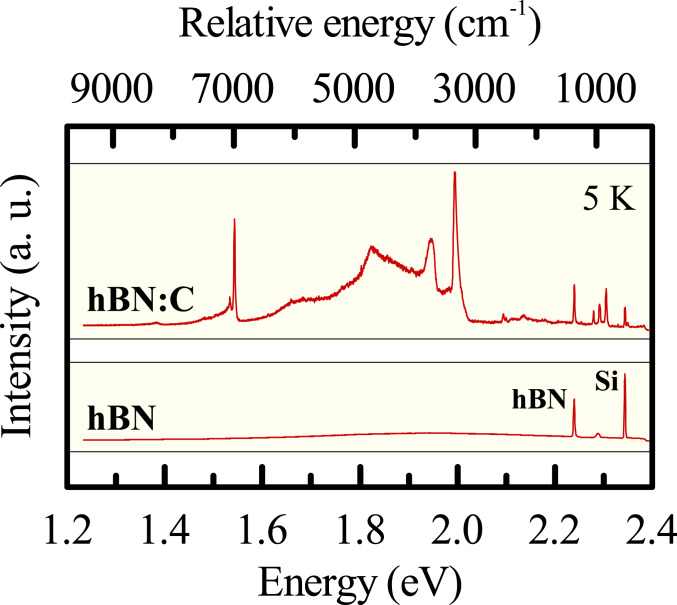
Low-temperature (5 K) micro-optical response of a pristine hBN film (lower spectrum) as compared to that of hBN:C film (upper spectrum) under 514.4-nm (2.41 eV) single-frequency DPSS laser excitation. The spectrum of the pristine hBN film is dominated by the conventional Raman-scattering response of hBN (phonon peak at 1,365 cm^−1^) and of the underneath silicon substrate (phonon excitation at 524 cm^−1^). The hBN:C films display much more complex optical spectrum with multiple photoluminescence resonances accompanying the two major Raman scattering lines seen for pristine material. a.u., arbitrary units.

C-doping is believed to introduce shallow and deep levels in hBN and has been recently reported to modify its photoluminescence spectra in the far-UV region (20). Here, we examine the optical response of C-enriched hBN in the visible and near-infrared spectral range. To start with their characterization, we inspected the low-temperature (5 K) microoptical response of multiple species of both pristine and hBN:C by exciting them with a 2.41-eV laser beam. Representative optical spectra are presented in Fig. 1. The dominant resonance observed for the pristine hBN films was due to an optical-phonon, Raman scattering peak at 1,365 cm^−1^. The optical response of the hBN:C species was, on the other hand, very rich. Let us note that the presented spectra were rather homogenous across the investigated samples. Multiple samples showed the same optical response, and the micro-optical mapping demonstrated that the intensity of emission correlates well with the thickness of the hBN:C film, but no qualitative differences were discerned. Selected maps may be found in *SI Appendix*, Fig. S1. This finding encourages the interpretation of the observed light emission as optical transitions in an ensemble of C-related defects in hBN materials. Such defect centers clearly introduce well-defined midgap levels that enable the radiative, intracenter recombination processes. We will proceed with further characterization of the resonances seen in the optical spectra of hBN:C films and discuss their possible origin, together with the assessment of the physical character of the emitting states.

## Franck–Condon Spectrum of a Defect Strongly Coupled to Optical Phonons

**Fig. 2.**
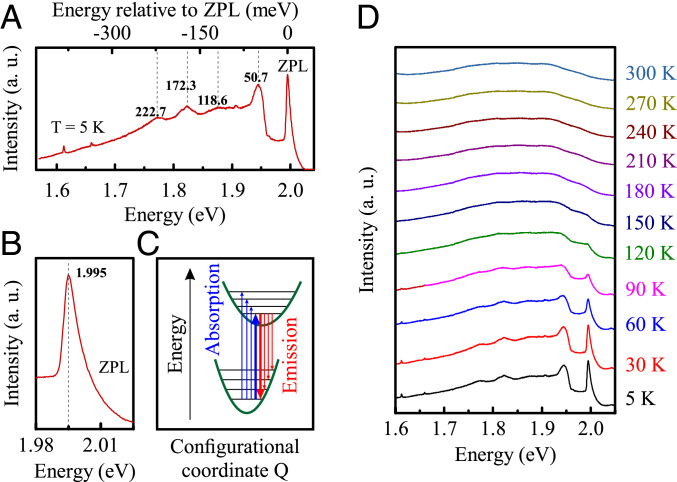
(*A*) The low-temperature spectrum of an hBN:C film under 2.41-eV laser excitation is presented in greater detail to demonstrate the existence of multiple resonances. (*B*) The highest-energy resonance, identified as a ZPL, is located at 1.995 eV and displays an asymmetric line shape. (*C*) This spectrum is interpreted in terms of Franck–Condon model with transitions between two electronic levels coupled to equidistant (in harmonic oscillator approximation) vibrational modes. The ZPL is emphasized by using thicker arrows to mark it in the scheme. (*D*) The evolution of the emission spectrum with temperature shows that the distinctive resonances are conspicuous exclusively at low temperature. The spectra are shifted vertically for better visibility. A.u., arbitrary units.

We firstly focused on the interpretation of the optical response of hBN:C in the energy range 1.6 to 2.05 eV. The resonances appearing therein, depicted in Fig. 2*A*, formed a pattern characteristic of a Franck–Condon type of spectrum (21, 22). When localized electronic levels are strongly coupled to optical phonons, photo-excitation elevates electrons between two molecular-like states while simultaneously annihilating (in absorption processes) or creating (in emission processes) phonons. Such a description has been found to accurately account for the optical transitions between defect states in many types of solid-state structures, the renowned example being a nitrogen-vacancy (NV) center in diamond (23–25). Notably, a Frank–Condon spectrum has been also identified in hBN, but in the near-UV spectral region (around 4.1 eV), and likely not related to a defect induced by C-doping (20). In the Franck–Condon picture, the highest energy resonance constitutes a transition without involvement of phonons, the so-called zero-phonon line (ZPL), which we observed at 1.995 eV. The magnified view of the ZPL is presented in Fig. 2*B* to illustrate the asymmetric shape of this resonance. Such a feature is usually attributed to a homogenous broadening associated with acoustic phonons (26, 27). That is, in a simple view, because processes with the annihilation of phonons are restricted to low-energy vibrational modes according to their thermal distribution, while the processes with the creation of phonons may involve modes of any energy. The ZPL resonance was accompanied by a low-energy phonon side band formed by vibronic resonances due to local* and/or quasilocal^†^ phonons coupled to the electronic states partaking in the optical transition. The absorption and emission processes that are relevant for the optical response of a defect strongly coupled to optical phonons and following the Franck–Condon model are schematically presented in Fig. 2*C*.

The fundamental figures of merit characterizing a Franck–Condon emitter are Debye–Waller (28, 29) and Huang–Rhys (30) factors, which are used to define and determine electron–phonon coupling strength. The Debye–Waller factor is calculated as the spectral weight of the ZPL with respect to the total emission intensity of the defect. For our emitters, we found the Debye–Waller factor (*w*) to be equal to 3.0%, which is comparable with the value obtained for other defect centers in wide-gap materials (25). The Huang–Rhys factor (*S*) is less straightforward to establish; hence, we used a phenomenological formula (31–34) *S* = −ln(*w*) to obtain an estimation of an average value of phonons emitted in a single recombination process that yielded, in our case, 3.5. Such a number appears to be reasonable based on the characteristics of the phonon side-band spectrum (Fig. 2*A*). It is formed by one well-pronounced resonance 50.7 meV below the ZPL, followed by several significantly weaker peaks. Let us note that the energy of the consecutive resonances does not correspond to multiple phonon replicas of an individual vibrational mode. One needs to consider at least two different phonons to account for four resonances marked in the figure. This observation may act as an indication that the defect levels partaking in the recombination process are efficiently coupled to more than one vibrational mode.

Further information about the optical properties of this defect center comes from the temperature dependence of the emission spectrum, which is presented in Fig. 2*D*. The distinctive resonances discernible in the lowest temperature spectrum were less apparent at higher temperatures, so that their evolution can be traced only up to 150 K. The broad emission band persisted up to room temperature, but the total integrated intensity decreased when increasing the sample temperature. Such behavior is understandable within the Franck–Condon model. The initial state of the recombination process, which is responsible for the emergence of the ZPL and the associated phonon replicas, depopulates at higher temperature in favor of higher-energy vibronic states. Therefore, the distinctive resonances, which are strictly tied to the population of initial state of the ZPL transition, become less pronounced at higher temperature. The decrease of the total emission intensity may be attributed to an activation of nonradiative processes.

## The 1.54-eV Emission Doublet and Associated Higher-Energy Transitions

**Fig. 3.**
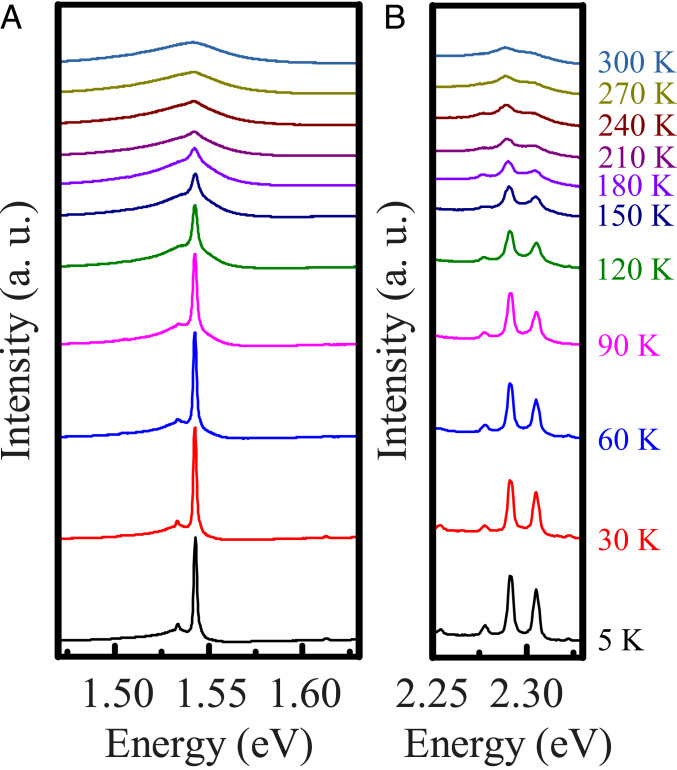
The impact of temperature on the 1.54-eV doublet of resonances (*A*) and a set of higher-energy narrow resonances (*B*) is presented. All of the lines in this energy range exhibit qualitatively identical evolution with temperature, as they persist up to room temperature while displaying significant broadening in form of asymmetric exponential-like tails, which accompany the main lines at higher temperature. Such observation inspires the attribution of these narrow lines to optical transitions within the same defect center between levels that couple to acoustic phonons. The spectra taken at various temperatures have been shifted vertically for enhanced clarity. a.u., arbitrary units.

The Franck–Condon model cannot account for the entirety of the emission spectrum that we observed for the hBN:C flakes. Another well-pronounced feature appeared as a doublet of resonances at 1.54 eV. As shown in Fig. 3*A*, these lines persisted up to room temperature. At low temperature, they took the form of narrow, distinctive resonances, which became accompanied by higher- and lower-energy shoulders when the temperature was increased. Such behavior may be expected of a defect that is effectively decoupled from optical phonons of the host lattice (e.g., due to a particular symmetry of the electronic states). Usually, the optical transitions may still involve, especially at higher temperature, creation and/or annihilation of acoustic phonons (35), as such processes do not require the strict selection rules.

An important example of a defect characterized by similar properties is a Cr^3+^ substitution for Al ions in corundum (Al_2_O_3_) crystals. Crystals with a substantial concentration of Cr^3+^ impurities (commonly known as ruby) display a robust photoluminescence in the form of a lower-energy doublet (R lines) accompanied by weaker, higher-energy transitions (B lines) arising due to transitions between intrinsic levels of Cr^3+^ ions (36). A very similar pattern was discernible in the optical response of hBN:C material. However, quantitative differences between the narrow-line emission resonances in hBN:C and ruby crystals were apparent. Most importantly, the splitting between the two components of the doublet was significantly larger in the former material and equal to about 10 meV. Such splitting may arise due to spin-orbit coupling and/or lattice distortions (37), which lift the degeneracy of states involved in the recombination process (38, 39). Higher-energy narrow lines were also visible, and their most pronounced representatives appeared around 2.30 eV. They displayed qualitatively identical temperature evolution (Fig. 3*B*) as the fundamental 1.54-eV doublet; hence, it is plausible that the higher-energy narrow resonances appeared due to transitions within the same defect center.

## Coexistence of Various Types of Defect Spectra in hBN:C

A C atom may create two simple impurity types in hBN: a substitution for boron (forming donor states) or a substitution for nitrogen (forming acceptor states). Their more complicated variations, such as C substitution for boron combined with adjacent nitrogen vacancy, have also been proposed as a possible origin of narrow-lines luminescence in hBN (40). Such defect centers give rise to a ground-state midgap level that may exist in various charge states. Transitions between different charge states could give rise to a Franck–Condon type of spectra, as was observed in our hBN:C samples.

However, an alien atom in the lattice structure introduces, in principle, an entire structure of levels formed by various configurations of its electrons on atomic valence shells. The intradefect transitions between these levels may give rise to narrow emission lines akin to those commonly observed in atomic spectroscopy. The resonances due to transitions between atomic-like levels may persist when atoms become impurities in crystals. The observation of such resonances gives insight into the alternation of the atomic-level structure caused by the crystal environment, as well as coupling of the electronic states with lattice motion. The emission doublet at 1.54 eV observed in hBN:C, accompanied by higher-energy transitions, exhibited characteristics of such intradefect transitions.

**Fig. 4.**
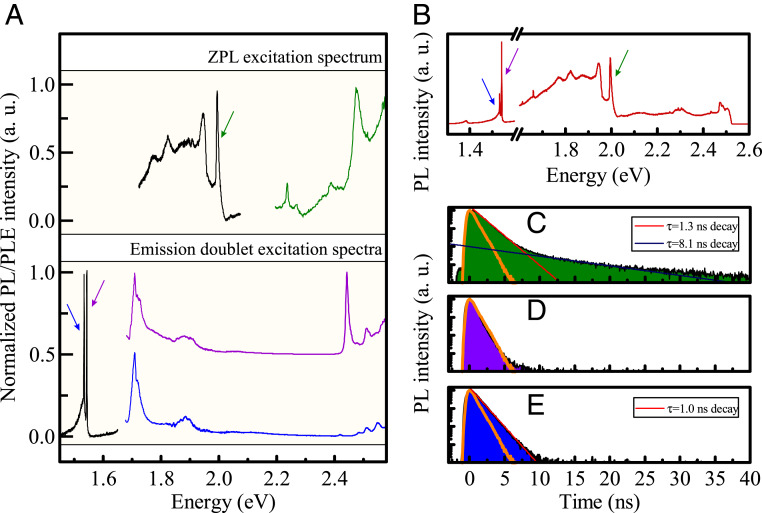
(*A*) PLE spectra measured at 4 K are presented for the Franck–Condon-type emitting center (*Upper*; green curve represents emission intensity of ZPL) and for the 1.54-eV emission doublet (*Lower*; purple and blue curves represent emission intensity of higher and lower energy resonances, respectively). The PL spectrum in *A*, *Upper* (black curve) is excited at 2.477 eV, and the PL spectrum in *A*, *Lower* (black curve) is excited at 2.095 eV. The spectra have been shifted vertically for clarity. The time-resolved study of emission dynamics was performed with a 437-nm (2.824-eV) picosecond laser. (*B*) The time-integrated low temperature (4 K) PL spectrum under such excitation is demonstrated. (*C*–*E*) All three fundamental transitions are visible, and the decay profiles are shown for ZPL (*C*), higher-energy resonance (*D*), and lower-energy resonance (*E*) of the 1.54-eV doublet. The orange transients in *C*–*E* represent the decay profile of the laser and are indicative of the lower limit of the measurable decay time. a.u., arbitrary units.

The possibility to observe both types of transitions strongly depends on two factors: the details of absorption spectra that determine the efficiency of occupying the appropriate emitting sates and the time scales of recombination (both radiative and nonradiative) and/or relaxation processes. We have investigated these aspects of our emitting centers by performing photoluminescence excitation (PLE) spectroscopy and measurements of emission-decay times. The PLE spectra for the ZPL of the Franck–Condon-type spectrum and both resonances forming the 1.54-eV doublet are presented in Fig. 4*A*.

The ZPL is efficiently excited by photons from a broad range of wavelengths. There is a general trend of increasing emission intensity for the higher energy of excitation, combined with the appearance of additional absorption resonances. In the Franck–Condon model, the resonant absorption may appear due to alignment of the energy of excitation photon with a ZPL transition energy enlarged by the energy of an appropriate phonon. Alternatively, the enhanced absorption may correspond to the existence of higher-energy electronic levels (e.g., of a different charge state of the defect). The major absorption resonance at 2.477 eV was energetically more displaced from the ZPL than a phonon-assisted transition would allow; hence, it is most likely of the latter type.

The 1.54-eV emission doublet clearly exhibited a resonant character of excitation mechanisms. When studying intradefect transitions, the resonances in absorption-like spectra usually correspond to higher-energy states of the defect, as is the case, e.g., for Cr^3+^ impurities of ruby (41). The PLE spectra of the 1.54-eV doublet unveiled prominent resonances, for which the emission intensity was over an order of magnitude stronger than in nonresonant regime. Both lines of the doublet shared a lower-energy resonance at 1.709 eV (about 0.17 eV above the emission energy). The higher-energy line of the doublet displayed also a higher-energy resonance at 2.443 eV, which was absent in the excitation spectrum for the lower-energy line. Such an observation suggests that there exists a splitting in the initial state of the recombination processes, which gives rise to the 1.54-eV emission doublet. The existence of a robust resonance at 2.443 eV is indicative of a presence of a higher-energy defect level, whose occupation may be transferred efficiently, through a selective relaxation process, only to the state that constitutes the origin of the higher-energy resonance of the 1.54-eV doublet. The observations based on the PLE spectra of the emission doublet may be summarized in the form of a Jablonski diagram (42) that shows the most pronounced atomic-like states of the defect together with corresponding transitions that are required for discerning the main features of the emission and absorption spectra (*SI Appendix*, Fig. S5).

Overall, the distinctive character of the excitation spectra clearly demonstrated that the efficiency of emission of a particular type of defect is strongly dependent on excitation conditions. Existence of robust resonances provides an opportunity to control which type of emission spectrum would be dominant in hBN:C samples.

Further information about the emission processes may be inferred from PL decay transients. These were measured for high-energy excitation (2.824 eV) with a 100-ps pulsed laser (see Fig. 4*B* for the PL spectrum measured under pulsed excitation, which shows qualitatively identical features as the PL spectrum under continuous-wave excitation). The decay profile for the ZPL of the Franck–Condon-type (Fig. 4*C*) defect showed a multiexponential character. As the ZPL appears on top of the phonon side band, we associated the short time component (with a characteristic time τ = 1.3 ± 0.2 ns) to the emission dynamics of the ZPL and the weaker, long time component (with a characteristic time τ = 8.1 ± 2.2 ns) to the phonon-assisted recombination that contributes to the side band. The dynamics of the 1.54-eV emission doublet (Fig. 4 *D* and *E*) was quite different. The lower-energy line showed a monoexponential decay with characteristic time τ = 1.0 ± 0.1 ns. The decay time of the higher-energy resonance was faster and fell below the measurable time in our set-up (≤730 ± 100 ps). The monoexponential character of the decay transient suggests that the emission process is unperturbed by the existence of intermediate states.

The nanosecond lifetimes of the carriers participating in the recombination processes, which give rise to the 1.54-eV doublet and the 1.995-eV ZPL, demonstrate that these qualitatively different emitting centers are capable of light emission with similar efficiency. The longer lifetime component observable at the energy of the ZPL provides an additional argument that the carriers occupying Franck–Condon states may recombine via many body processes, e.g., involving creation of phonons.

## Summary

We have presented a comprehensive study of the optical response of C-enriched hBN crystals. We have found the emission signatures of two types of emission centers that display properties with many parallelisms to other well-known defect centers in solids, such as NV centers in diamond or Cr^3+^ impurities in ruby crystals. Through the combination of excitation and time-resolved spectroscopy, we have unveiled an intricate energy structure of the emitting defect centers and proposed a plausible interpretation of the recombination processes. Our findings demonstrate that C-related defects in hBN offer the possibility to study fundamental defect physics in two-dimensional (2D) crystals, which are compatible with constantly evolving and improving van der Waals technology. The successful demonstration of creating well-defined defect levels may inspire similar efforts to introduce various dopants into other compounds of the family of 2D materials. The properties of our defect centers strongly support and promote the notion that hBN is a material suitable for a variety of opto-electronic applications, including devices operating in visible and near-infrared spectral range.

## Methods

### Crystal Growth.

Single crystals of hBN were grown by the temperature-gradient method under high-pressure and high-temperature conditions. Colorless and transparent single crystals of hBN were obtained (43). To achieve C-doping, selected crystals were annealed for 1 h at 2,000 °C with nitrogen gas flow by using high-frequency furnace with graphite susceptor.

### Sample Preparation.

Thin films of pristine hBN and hBN:C were isolated by mechanical exfoliation and deposited onto ultraflat silicon substrates cleaned by plasma ashing. A common technique of two-step exfoliation based on polydimethylsiloxane films was used. The samples were inspected in an optical microscope in order to locate individual flakes, whose thickness could be estimated by their optical contrast with respect to the substrate.

### Optical Spectroscopy.

The optical spectra were measured in a confocal microspectroscopy set-up. A cold-finger cryostat was used to cool down the samples to 4 K, and a resistor-based heater allowed control of the temperature. A long working-distance objective (50×) and a set of appropriate filters/polarizers were employed to achieve focalization of a laser beam down to a spot of about 1-μm diameter and collection of light from the sample. A 500-cm spectrometer with a charge-coupled device camera provided spectral resolution and light-detection capabilities. The emission spectra were measured by using 514.4-nm narrow-linewidth diode pumped solid-state (DPSS) laser. The excitation spectroscopy was realized with a broad-band supercontinuum light source coupled to a spectrometer to achieve tunable monochromatic (linewidth of about 2 nm) excitation. A 100-ps, 437-nm laser was used to perform time-resolved measurements. An avalanche photodiode (APD) counted the photons to obtain PL decay profiles. By measuring the laser pulses directly with the APD (orange curves in Fig. 4 *C*–*E*), we established the lowest measurable decay time to be 730 ± 100 ps. The decay profiles were fitted with exponential profiles:

$$I\left(t\right)=I_{0}*exp\left(-\frac{t}{\tau }\right),$$

where *I*_0_ is a multiplicative constant, and τ is the characteristic decay time.

The measurements of PL spectra in a magnetic field (presented in *SI Appendix*, Fig. S6) were done in a superconducting magnet coupled to a helium-bath cryostat. A fiber-based probe hosting a miniaturized optical table with a set of lenses, mirrors, and filters was used to focalize the laser and collect emitter light. Piezo-stages with *x*–*y*–*z* motion capabilities were used to position the sample. The sample was cooled down via helium-exchange gas.

### Data Availability.

All of the supplementary data on the optical characterization of the hBN:C films are provided in *SI Appendix*, Figs. S1–S6.

## Supplementary Material

Supplementary File

## Footnotes

*Lattice oscillatory modes related to movement of atoms in the close neighborhood of the defect, hence creating vibration states highly localized in the real space.

^†^Lattice vibrations composed of modes that are delocalized in real space, but whose contribution to the phonon spectrum is altered by the presence of the defect and effectively leading to an appearance of a phonon-like resonance.
