# Supplementary material for: Midgap radiative centers in carbon-enriched hexagonal boron nitride
Source: Proc Natl Acad Sci U S A. 2020 Jun 1;117(24):13214–9. doi: 10.1073/pnas.2003895117 (PMC7306815; doi:10.1073/pnas.2003895117)
Supplement: Supplementary File [file pnas.2003895117.sapp.pdf]

Supplementary Information: *Mid-gap radiative centres in carbon enriched hexagonal boron nitride*

Maciej Koperski[1,*], Diana Vaclavkova[2], Kenji Watanabe[3], Takashi Taniguchi[3], Kostya S. Novoselov[1†], Marek Potemski[2,4,‡]

[1] Department of Materials Science and Engineering, National University of Singapore, 117575, Singapore

[2] Laboratoire National des Champs Magnétiques Intenses, CNRS-UGA-UPS-INSA-EMFL, 25 avenue des Martyrs, 38042 Grenoble, France

[3] National Institute for Materials Science, Tsukuba, Ibaraki, 305-0044, Japan

[4] Institute of Experimental Physics, Faculty of Physics, University of Warsaw, ul. Pasteura 5, PL-02-093 Warsaw, Poland

1. Spectral mapping of pristine and carbon-doped hBN.

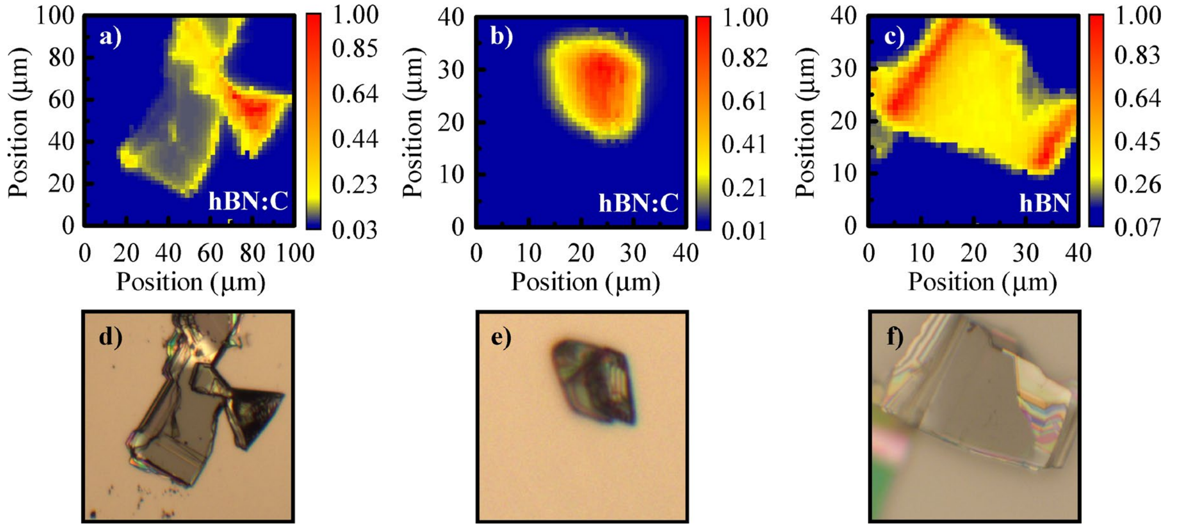

Figure S1. The micro-optical intensity colour-maps under 514.4 nm excitation are presented for hBN:C flakes **(a,b)** and hBN flake **(c)**. For the carbon-doped hBN films the intensity of zero-phonon line of the Franck-Condon emitter at 1.995 eV is monitored when scanning the focused laser beam across the sample's surface. For the pristine hBN flake the intensity of the Raman peak at 1365 cm^{-1} is used. The optical images of the flakes investigated in the mapping experiments are demonstrated **(d-f)**.

The mapping experiments are used to study the homogeneity of the optical response of the hBN:C films. The micro-photoluminescence maps of two selected specimen are presented in **Fig. S1(a,b)**. The optical spectrum, shown if **Fig. 1** of the main text, comes from the flake depicted in **Fig. S1(a)**. It is observed that the character of the emission spectrum in terms of appearance of

* e-mail: msemaci@nus.edu.sg
† e-mail: kostya@nus.edu.sg
‡ e-mail: marek.potemski@lncmi.cnrs.fr

particular resonances and their energy is uniform at various locations of the same flake and between different flakes. Consequently, the micro-photoluminescence maps do not display any discernible differences when different resonances are used for monitoring the intensity of emission. A micro-optical map of a pristine hBN flake is presented in **Fig. S1(c)**. In this case, the intensity of the 1365 cm^{-1} hBN Raman resonance is plotted. The mapping experiments, when analysed comparatively with similar data for hBN crystals from various sources(1), provide information that no signatures of defect-related emission may be easily found in our pristine crystals that have not been subjected to annealing in a graphite furnace. For comparison and estimation of the film thicknesses of exfoliated flakes based on optical contrast, optical images of the flakes are presented in **Fig S1 (d-f)**. The studied flakes are characterised by a broad range of thicknesses from tens to hundreds of nanometres. Based on the comparison between the micro-photoluminescence maps and optical images one can conclude that there exists a correlation between the intensity of emission and the film thickness. This observation indicates that the defect density is uniform so that thicker parts are hosting a larger number of emitting defect centres.

2. Power dependence of the emission intensity of ZPL and 1.54 eV doublet resonances.

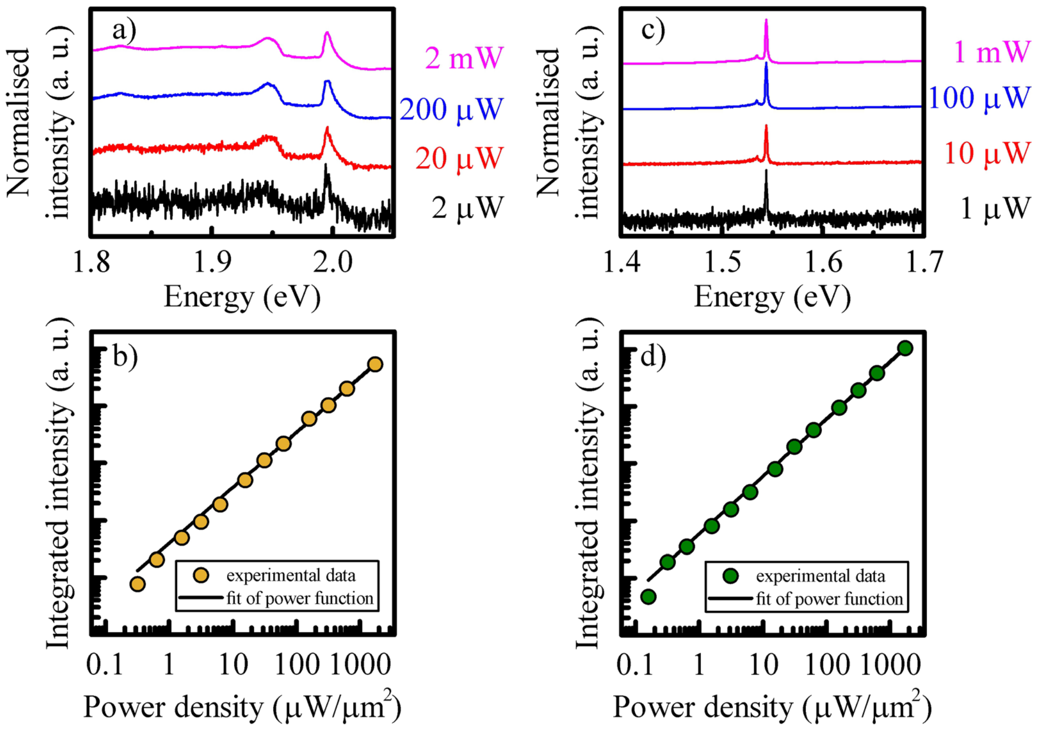

Figure S2. The low temperature (5 K) power-normalised spectra of the ZPL **(a)** and 1.54 eV doublet **(c)** are presented for selected values of excitation power spanning 3 orders of magnitude in **(a)** and **(c)** and 4 orders of magnitude in **(b)** and **(d)**. The spectra are obtained for 514.4 nm laser excitation with the beam focused to a spot of about 1 μm diameter. The integrated intensity of the ZPL **(b)** and the higher energy line of the 1.54 eV doublet **(d)** is demonstrated as a function of power density on the sample. In the studied regime of excitation power, a linear increase of the emission intensity for both investigated types of emission resonances is observed without any evidence of saturation.

The investigation of the emission intensity with varying excitation power provides information about processes and mechanisms responsible for delivering photo-excited carriers to the

emitting states. We have studied the evolution of the integrated weight of the emission resonances for the ZPL and 1.54 emission doublet across over 4 orders of magnitude of excitation power. As demonstrated in **Fig. S2**, the major characteristics of the Franck-Condon emitter and 1.54 eV emission doublet are unperturbed by varying excitation power. The emission intensity of the ZPL and the higher energy line of the doublet displays a linear dependence on the excitation power, as quantitatively verified by fitting the data with a power function:

$$I(P) = I_0 * P^{\alpha}$$

where I_0 is a multiplicative constant and α is the power coefficient. We have found $\alpha = 1.02 \pm 0.02$ for the ZPL and $\alpha = 1.03 \pm 0.02$ for the higher energy line of the 1.54 doublet to provide the best fit in terms of mean square error. A linear power dependence of the emission intensity is characteristic of intra-defect transitions between atomic-like states, further supporting our interpretation of the emission resonances in hBN:C films. Alternative emitting centres, involving formation of excitonic states, often allow formation of molecular-like states (biexcitons and other multi-exciton complexes). Those may result in superlinear dependence of the occupation of those states on the excitation power as well as induce more complex structure of levels and dynamics of interstate transitions.

The absence of saturation (up to about of 5 mW of laser power focused on the sample) for both investigated emission resonances may be related to the regime of below band-gap excitation. The PLE data unambiguously demonstrates that the absorption processes have strongly resonant character, so that the enhanced absorption corresponds to higher energy states of the defect. For that reason, it is plausible to assume that the major channels of excitation, responsible for appearance of our resonances, is related to the on-site absorption of light by the defects. Therefore, the effective power of light that is absorbed by the hBN:C films may be in fact significantly lower than the total power that is measured in the experiments and is likely to scale with the defect density.

3. Polarisation properties of the 1.54 eV doublet and ZPL resonances.

As we are looking at emission properties of an ensemble of defects, the linear polarisation properties will be inherently averaged over multiple individual emitting centres. For that reason, we investigate the degree of linear polarisation at 2 locations characterised by significantly different emission intensity. We analyse this data while making an assumption that the emission intensity correlates with the number of defects that contribute to the light collected in our set-up. In **Fig. S3** we present the spectra detected in linear polarisation along mutually perpendicular axes that correspond to the opposite extrema of emission intensity as seen in a polar plot.

The ZPL and the 1.54 eV doublet of resonances show a certain degree of linear polarisation at both locations. We analyse the angular dependence of the emission intensity by fitting the data with a squared sinusoidal function, as the probability of a linearly polarised photon passing through the detection setup is proportional to the component of the oscillating electric field projected onto the axis of the polarizer:

$$I(\alpha) = I_0 + A * \left(sin(\alpha + \alpha_0)\right)^2$$

where I_0 is a constant that is indicative of minimum intensity, A is the amplitude of oscillations and α_0 is a phase shift with respect to arbitrarily chosen axis of the polarizer. As we normalise our spectra so that the maximum intensity is equal to 1 (i. e., $I_0 + A = 1$), the polarisation degree is given by:

$$P = \frac{I_{max} - I_{min}}{I_{max} + I_{min}} = \frac{A}{2 - A}$$

We have found $P = 8.6$ % for the ZPL and $P = 30.6$ % for the higher energy line of the doublet at 1st location and $P = 32.2$ % for the ZPL and $P = 59.7$ % for the higher energy line of the doublet at 2nd location. It is apparent that the polarisation degree at the location with smaller number of emitting defect centres is significantly larger. Such observation suggests that individual defect centres may be fully linearly polarised (just like, e. g. single photon emitters in hBN(1, 2)) with a characteristic axis oriented along different directions, so that in ensembles of emitters the degree of polarisation is smaller and the effective polarisation axis defined by an averaged contribution of the individual emitting centres. As the orientation of the effective axis is different between the two studied locations (for the same alignment of the polarizer), we can rule out the possibility that the linear polarisation in inherited from linearly polarised excitation photons (i. e., optical pumping mechanisms are not very efficient under excitation conditions used for this study).

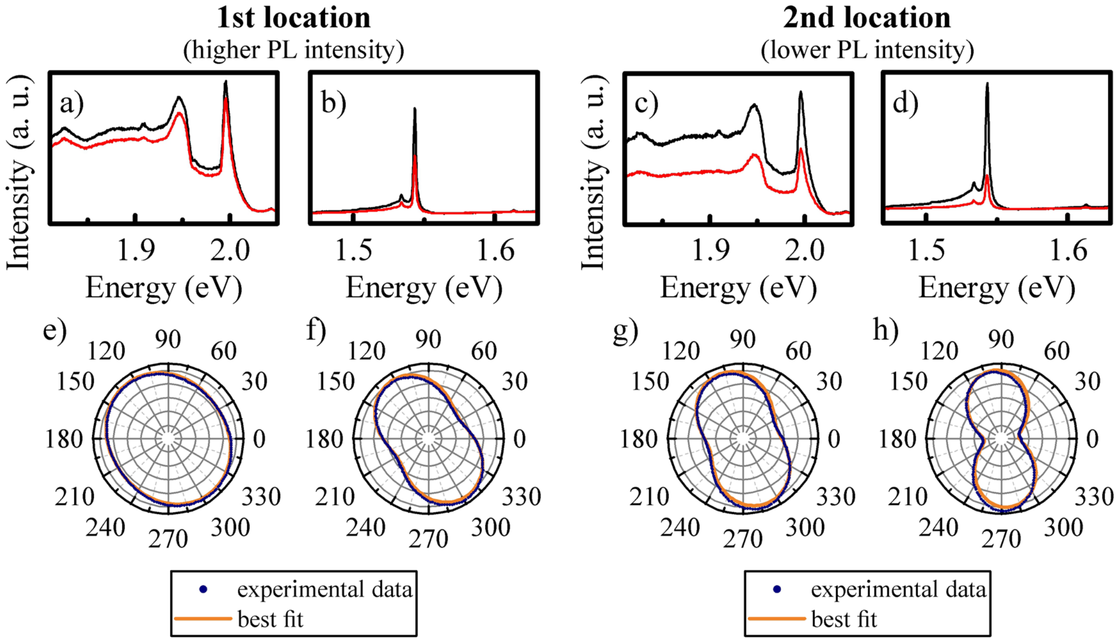

Figure S3. The low temperature (5 K) emission spectra detected with linear polarisation resolution at two mutually perpendicular angles corresponding to highest (black curve) and lowest (red curves) emission intensity of particular resonances: ZPL **(a, c)** and emission doublet **(b, d)**. Two location on different exfoliated hBN:C flakes are inspected: one with higher total emission intensity (1st location) and the other with lower emission intensity (2nd location). The systematic dependence of the emission intensity on the linear polarisation angle was measured by rotating a λ/2 waveplate located in front of a linear polarizer. The data for the ZPL and higher energy of the 1.54 eV doublet is presented for both locations in form of polar plots **(e-h)**. 514.4 nm laser excitation with a power of 1 mW was used to obtain the polarisation data for both locations on two flakes on the same substrate, so that the excitation conditions, regarding the energy and power, are comparable.

4. Asymmetry of the ZPL.

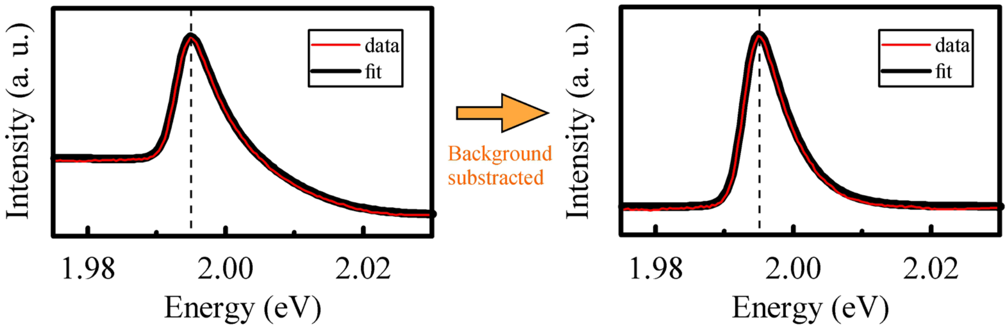

Figure S4. The resonance corresponding to the zero-phonon line of the Franck-Condon emitter in hBN:C films is presented in finer detail **(a)**. The resonance line (red curve) has been fitted by a phenomenological asymmetric peak function (black curve) with exponentially decaying lower and higher energy tails. A background function was introduced to account for a phonon sideband background that overlaps the zero-phonon line resonance. The spectrum with the background subtracted **(b)** offers a more explicit view of the asymmetric character of the resonance.

The line shape of the zero-phonon line of a Franck-Condon type of emitter is indicative of the homogenous and inhomogeneous broadening due to the impact of the crystal environment on the character of electronic states, which partake in the optical transition(3, 4). One of the important aspects of introducing a defect into a crystal structure is its coupling with long-wavelength acoustic phonons. Due to long-range character of interactions involving acoustic vibrational modes, they may be relevant for designing mechanisms of coupling between distant defect centres.

The theoretical description of the line shape characterising a phonon-mediated optical transition between two electronic levels is possible within the Einstein or Debye model. However, due to complexity of phonon spectra in realistic systems, these models are not easily applicable. Therefore, we will take a phenomenological approach to inspect the shape of the zero-phonon line at 1.995 eV (see **Fig. S4(a)**). It has been fitted by an asymmetric peak function with exponentially decaying tails:

$$I(E) = I_{BG}(E) + I_0 * \left(\frac{1}{1 + exp\left(-\frac{E - E_0 + {}^{\varepsilon_1}/_2}{\varepsilon_2} \right)} \right) * \left(1 - \frac{1}{1 + exp\left(-\frac{E - E_0 - {}^{\varepsilon_1}/_2}{\varepsilon_3} \right)} \right)$$

Two constants, ε_1 and ε_2, constitute the characteristic energy scale of the low-energy and high-energy tails of the resonance, respectively. We have found the values of $\varepsilon_2 = 1.0 \pm 0.1$ meV and $\varepsilon_3 = 4.0 \pm 0.2$ meV to provide the best fit in terms of mean squared error. These parameters allow us to quantify the asymmetry of the resonance as $\varepsilon_3/\varepsilon_2 = 4.0$. E_0 is the energetic position of the resonance and ε_1 is indicative of the width of the resonance. In order to account for the lower energy phonon sideband, which overlaps with the ZPL, we have introduced a generic background tailored to the shape of the band in the vicinity of ZPL:

$$I_{BG}(E) = I_C + \left(1 + tanh\left(-\gamma * \left(E + E_{0,BG}\right)\right)\right)/\alpha$$

I_C is an energy-independent contribution, $E_{0,BG}$ is a parameter that defines the energy position of the slope, γ determines the slope's steepness and α is a normalisation constant. The subtraction of the background function allows us to present the ZPL resonance in a more evident way (**Fig. S4(b)**).

5. Jablonski diagram of the 1.54 eV emission doublet.

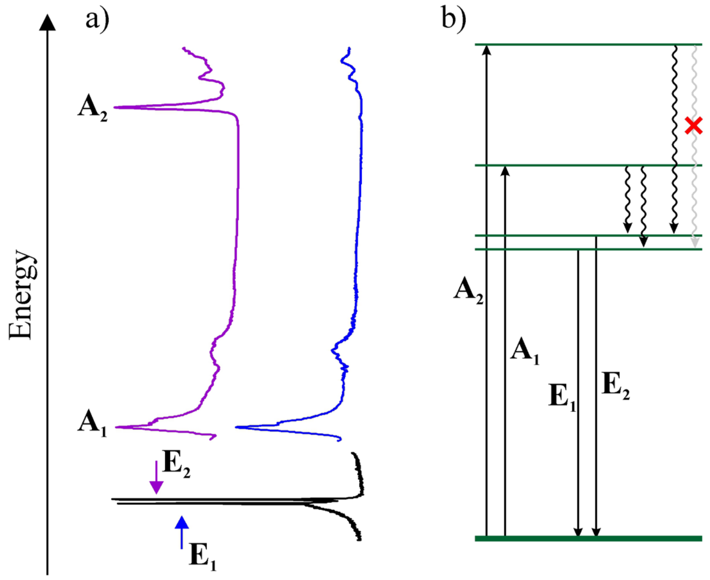

Figure S5. An overview of the photoluminescence (black curve) and quasi-absorption spectra of the 1.54 eV emission doublet are presented **(a)**. The blue/purple curves corresponding to the lower/higher energy resonances of the doublet, respectively. These data may be used to form a Jablonski diagram **(b)** based on the fundamental defect states, which give rise to the most pronounced emission resonances (**E_1** and **E_2**) and absorption resonances (**A_1** and **A_2**). The radiative and non-radiative transitions, necessary to account for the main features of the absorption and emission spectra, are marked with arrows.

The comparative analysis of the emission and absorption spectra is a powerful tool to unveil the energy levels of an emitting defect centre(5). The efficiency of radiative and non-radiative transitions between these levels may also be qualitatively appraised. We performed such analysis for the fundamental 1.54 eV doublet of resonances (**Fig. S5(a)**) in the main text, that allows us to illustrate the energy landscape of this defect centre in form of a Jablonski diagram(6) (**Fig. S5(b)**). Such chart may provide information about the mid-gap energy levels introduced by an impurity in the crystal lattice, which originate from atomic-like states altered by interactions with the crystal environment (crystal field, strain, lattice motion, etc.). This knowledge is crucial for developing an understanding of the fundamental physical properties of the defect. It is also relevant from the point of view of practical aspects that require efficient and selective excitation of a particular intra-defect transitions.

The presented diagram illustrates the minimal number of states that are needed to account for emission resonances **E_1** and **E_2** and major absorption resonances **A_1** and **A_2**. However, more detailed fine structure may be hinted in our spectra. The higher energy shoulder displayed by resonance **A_1** can be a signature of a splitting in the ground state of the defect (i. e., the final state in the processes **E_1** and **E_2**). Additional features of these spectra, such as a broader resonance between **A_1** and **A_2** as well as weaker resonances above resonance **A_2** may be indicative of the existence of

additional states that are characterised by smaller oscillator strength of associated optical transitions. Let it also be noted that the weaker emission resonances at 2.3 eV presented in **Fig. 3(b)** of the main text, that we associated with the optical transitions within the same defect centre, appear 0.153 eV below the **A_2** resonance, similarly as resonances **E_1/E_2** appear 0.170 eV below **A_1** resonance. Therefore, it is plausible that additional emitting states exist below the **A_2** resonance.

6. Magneto-optical spectra of hBN:C films.

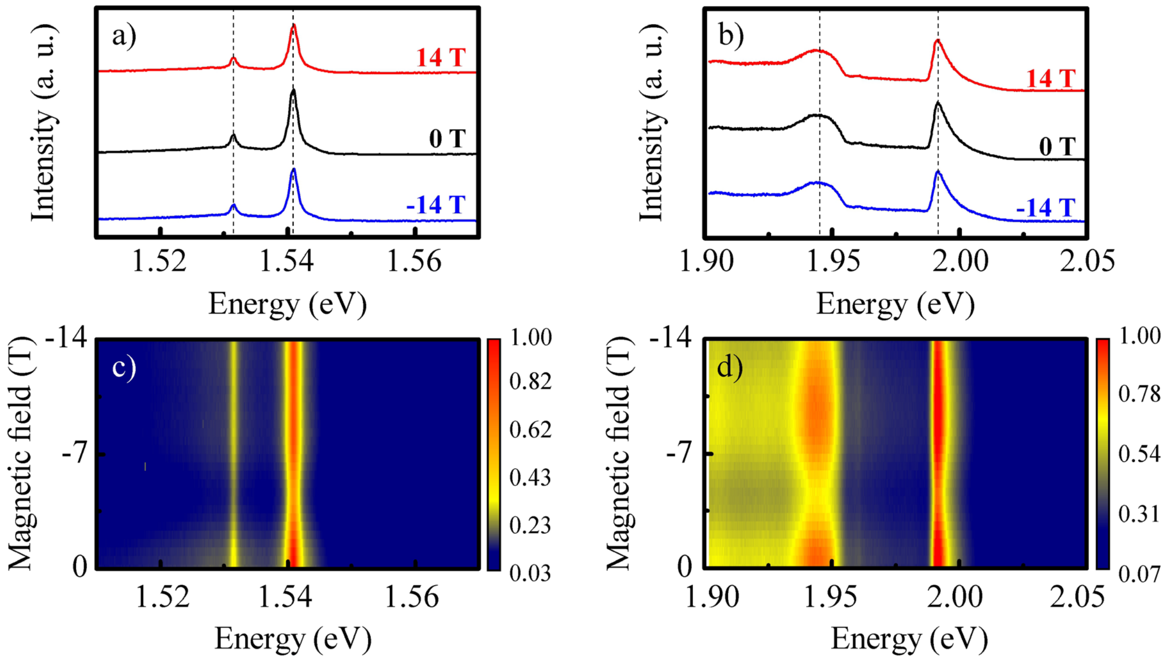

Figure S6. The magneto-optical spectra (shifted vertically) of the 1.54 eV doublet of resonances **(a)** and the ZPL accompanied by a resonance in the phonon sideband **(b)** are presented. The spectra are measured in Faraday configuration with the magnetic field applied perpendicularly to the surface of the hBN:C flakes (i. e. along *c*-axis of the hBN crystal). A combination of a $\lambda/4$ waveplate and a polariser is used to detect light in fixed circular polarisation and the magnetic field is applied in two polarities in order to observe both helicities. Colour maps **(c,d)** demonstrate systematically the evolution of the spectra in the magnetic field in the energy range corresponding to the presented spectra (the oscillations of the intensity arise from the Faraday effect due to injection of linearly polarised light into optical fibers).

Introduction of a defect into a crystal structure may form multiple states that are characterised by particular quantum numbers. From the point of view of optical transition, angular momentum plays a crucial role in determining, which states may radiatively recombine. According to first-principle calculations and predictions, carbon related impurities in hBN may give rise to levels with vanishing and/or integer angular momentum. Hence, nonmagnetic states as well as singlets and triplets may be expected to arise.

The differential g-factor* between two levels partaking in an optical transition may be studied directly by observing the evolution of emission resonances in a magnetic field with circular polarisation resolution. We performed such experiments, applying magnetic field along *c*-axis of hBN

* A g-factor of an individual level may be defined as $g\mu_B B = E_{B\uparrow} - E_{B\downarrow}$, where μ_B is Bohr magneton, B is the value of a magnetic field and $E_{B\uparrow/\downarrow}$ is the energy of the level when the magnetic field is applied in opposite directions ($\uparrow$ and $\downarrow$).

crystals, for both the emission doublet at 1.54 eV (**Fig. S6(a,c)**) as well as zero-phonon line and major resonance in the phonon sideband related to the Franck-Condon emitter (**Fig. S6(b,d)**). For all these resonances we observe no measurable splitting, hence we have to conclude that the $g_{\parallel c} \approx 0$ for all presented resonances. Such observation is in agreement with a vanishing g-factor of narrow lines emitting centres in hBN powder and exfoliated films(1). However, this finding remains puzzling, as a photon carries angular momentum equal to 1, which needs to be accounted for in the optical transition. It is plausible that the angular momentum is preserved via more complicated emission processes, e. g. involving recombination with emission of phonons. Our experiments also do not explore the possibility that the angular momentum would be aligned in the plane(7) of hBN layers (perpendicular to the *c*-axis).

References

1. M. Koperski, K. Nogajewski, M. Potemski, Single photon emitters in boron nitride: More than a supplementary material. *Opt. Commun.* **411**, 158-165 (2018).
2. T. T. Tran, K. Bray, M. J. Ford, M. Toth, I. Aharonovich, Quantum emission from hexagonal boron nitride monolayers. *Nat. Nanotechnol.* **11**, 37 (2016).
3. A. Kiel, Temperature-dependent linewidth of excited states in crystals. I. Line broadening due to adiabatic variation of the local fields. *Phys. Rev.* **126**, 1292 (1962).
4. R. H. Silsbee, Thermal broadening of the Mössbauer line and of narrow-line electronic spectra in solids. *Phys. Rev.* **128**, 1726 (1962).
5. J. A. Calviello, E. W. Fisher, Z. H. Heller, Direct $2T_1$-2E phonon relaxation in ruby and its effect upon R-line breadth. *J. Appl. Phys.* **37**, 3156 (1966).
6. A. Jablonski, Efficiency of anti-stokes fluorescence in dyes. *Nature* **131**, 839-840 (1933).
7. S. Sugano, Y. Tanabe, The line spectra of Cr-3+ ion in crystals. *Discussions of the Faraday Society* **26**, 43-48 (1958).
